# Endoscopy-assisted surgical management for a giant axillary cystic lymphangioma: a case report

**DOI:** 10.3389/fsurg.2025.1702543

**Published:** 2025-11-06

**Authors:** Hai-wei Chen, Rong-jiang Li, Yan-bang Niu, Xiong Lu

**Affiliations:** 1 Department of General Surgery, Baoji People’s Hospital, Baoji, Shaanxi, China; 2 Department of Radiology, Baoji People’s Hospital, Baoji, Shaanxi, China

**Keywords:** axilla, cystic lymphangioma, case report, endoscopy-assisted, surgery

## Abstract

A cystic lymphangioma is a benign malformation that arises from aberrant lymphatic development and commonly occurs in the neck and axilla. While typically asymptomatic, complete surgical excision remains the primary treatment. We present a case of a giant, left axillary cystic lymphangioma (approximately 15 cm × 15 cm × 10 cm) in a young female patient. The following endoscopy-assisted surgical approach was employed: a small incision facilitated initial access and establishment of the operative field, while endoscopy provided magnified visualization of the anatomical relationships between the mass and surrounding structures, enabling precise dissection and complete resection. Histopathological examination confirmed the mass to be a cystic lymphangioma. At the 6-month follow-up, the patient exhibited no complications or recurrence. No reports exist on endoscopic management. The successful management of a cystic lymphangioma in this case offers a valuable reference for the clinical treatment of similar lesions.

## Introduction

A cystic lymphangioma is an uncommon congenital benign tumor composed of lymphatic endothelial cells, with an incompletely understood pathogenesis (1, 2). The prevailing theory suggests that it results from abnormal embryonic lymphatic development, where sequestered primitive lymphatic sacs fail to establish communication with central venous drainage systems, leading to isolated lymphatic channels or sac proliferation (3). The common sites include the neck, axilla, mediastinum, subdiaphragmatic region, retroperitoneum, and mesentery (4). Axillary lymphangiomas, constituting approximately 20% of cases (5), carry a significant complication rate due to their proximity to the brachial plexus and subclavian vessels. Surgical excision is the treatment of choice (6, 7). All previously reported surgical approaches for axillary lymphangiomas involve open excision. This report details the first documented case of endoscopy-assisted resection of a giant axillary cystic lymphangioma.

## Case presentation

**Figure 1:**
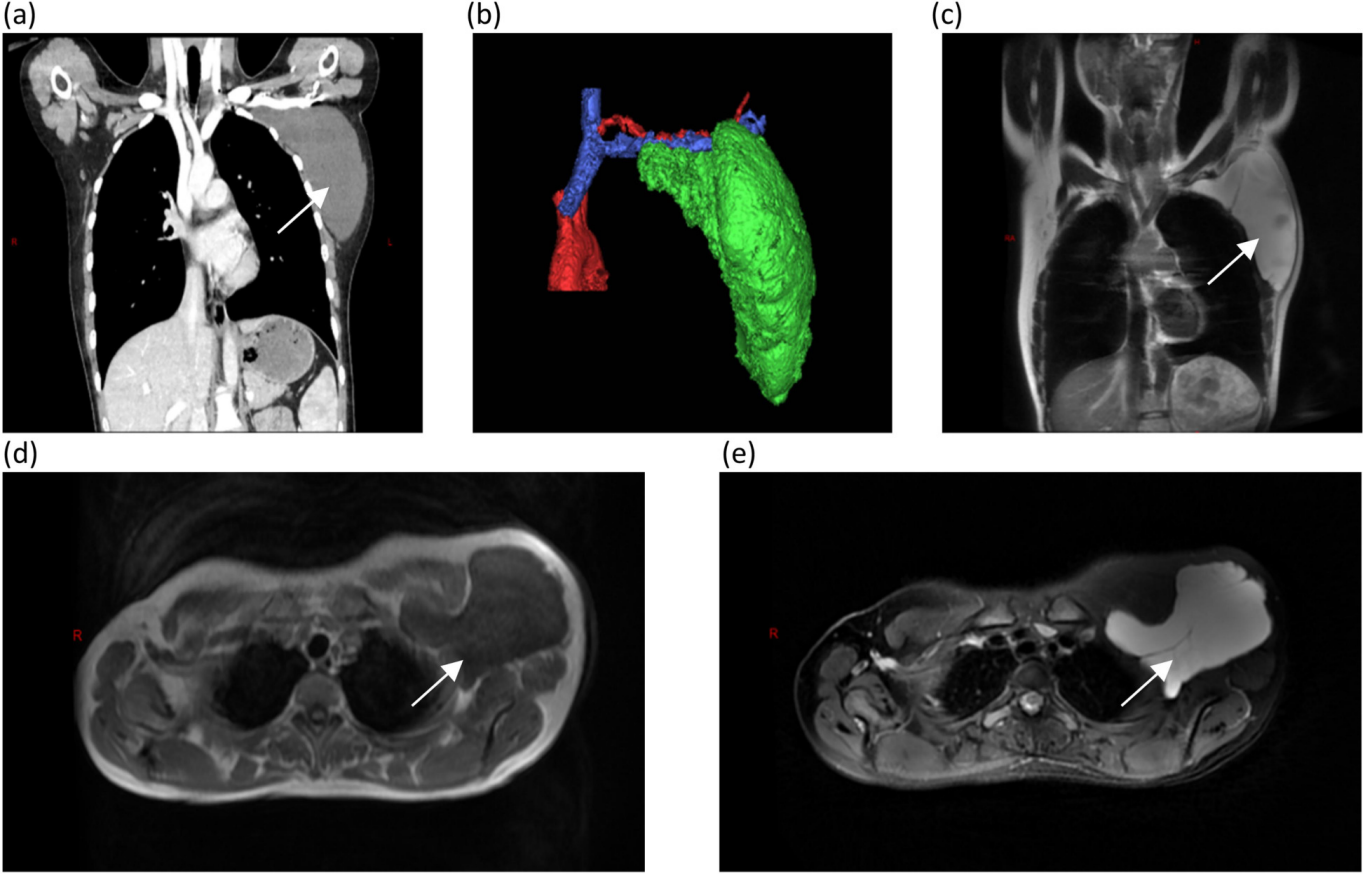
Computed tomography and magnetic resonance imaging, with arrows identifying the mass: **(a)** CTA revealing a large cystic mass in the left axilla; **(b)** A three-dimensional reconstruction showing that the mass is adjacent to the subclavian vessels; **(c)** A coronal view of an MRI examination; and **(d,e)** long T1 and T2 signals appear hypointense (dark) on T1-weighted images appearing hyperintense (bright) on T2-weighted images.

A 17-year-old female patient presented with a chief complaint of a left axillary mass persisting for over 1 month. Past medical history included sclerotherapy for a right facial hemangioma 8 years prior. She denied other illnesses, trauma, smoking, or alcohol use, and her menstrual history was regular. There was no family history of lymphangioma. Her vital signs upon admission were stable. A physical examination revealed normal development and moderate nutrition. The cardiopulmonary and abdominal exams were unremarkable. The local examination identified a 15 cm × 15 cm × 10 cm smooth, non-erythematous, non-ulcerated, soft mass in the left axilla. The mass extended superiorly to the axillary apex, inferiorly to the level of the seventh rib along the posterior axillary line, laterally to the lateral border of the left scapula, and medially beneath the medial third of the left clavicle. It was non-tender, relatively well-defined, and demonstrated limited mobility. Left upper extremity motor and sensory functions were intact. Laboratory investigations were within normal limits. Ultrasonography revealed a well-defined, irregular anechoic area with multiple septations and no significant internal vascularity, suggestive of a lymphatic origin. Computed tomography (CT) and CT angiography (CTA) demonstrated an irregular, non-enhancing, hypodense lesion (CT value 15 HU) in the left axilla, consistent with a cystic lymphangioma. Magnetic resonance imaging (MRI) showed a well-circumscribed, irregular mass exhibiting long T1 and long T2 signal intensity with internal linear short T2 septations, confirming a giant cystic lymphangioma (Figure 1). Aspiration yielded clear, pale yellow fluid, and the cytopathological analysis revealed abundant lymphocytes.

## Diagnosis and differential diagnosis

Based on the clinical presentation and imaging findings, a preoperative diagnosis of a giant, left axillary cystic lymphangioma was established. The differential diagnoses primarily include a cavernous hemangioma, soft tissue sarcoma, metastatic carcinoma, branchial cleft cyst, hematoma, and seroma (8–10). Table 1 summarizes the key distinguishing features.

**Table 1:** Differential diagnoses of cystic axillary masses.

Diagnosis	Clinical features	Imaging features	Pathological/immunohistochemical features
Cystic lymphangioma	Soft, slow-growing, often asymptomatic mass; may transilluminate	Water-density cyst (CT); homogeneous long T1 and T2 signals (MRI)	Cystic spaces lined by flattened endothelium; D2-40 (+), CD34 (−), WT1 (−)
Cavernous hemangioma	Soft, compressible mass; may have bluish discoloration	Heterogeneous mass with phleboliths possible; strong contrast enhancement	Blood-filled vascular spaces; CD34 (+), D2-40 (−)
Soft tissue sarcoma	Firm, rapidly growing mass; may be fixed to deep structures	Infiltrative, heterogeneous solid mass with necrosis and enhancement	Malignant spindle or round cells; specific IHC depends on sarcoma type
Metastatic carcinoma	History of malignancy; firm, fixed nodes	Cystic necrosis within lymph nodes is common	Malignant epithelial cells; cytokeratins (+), markers specific to the primary site
Branchial cleft cyst	Typically in the neck; rare in the axilla	Well-defined cystic lesion	Lined by squamous/columnar epithelium; lymphoid stroma in the wall
Hematoma/seroma	History of trauma or surgery; may resolve over time	Variable appearance (acute to chronic); no internal vascularity	Blood products or serous fluid; no epithelial lining

## Surgical management

**Figure 2:**
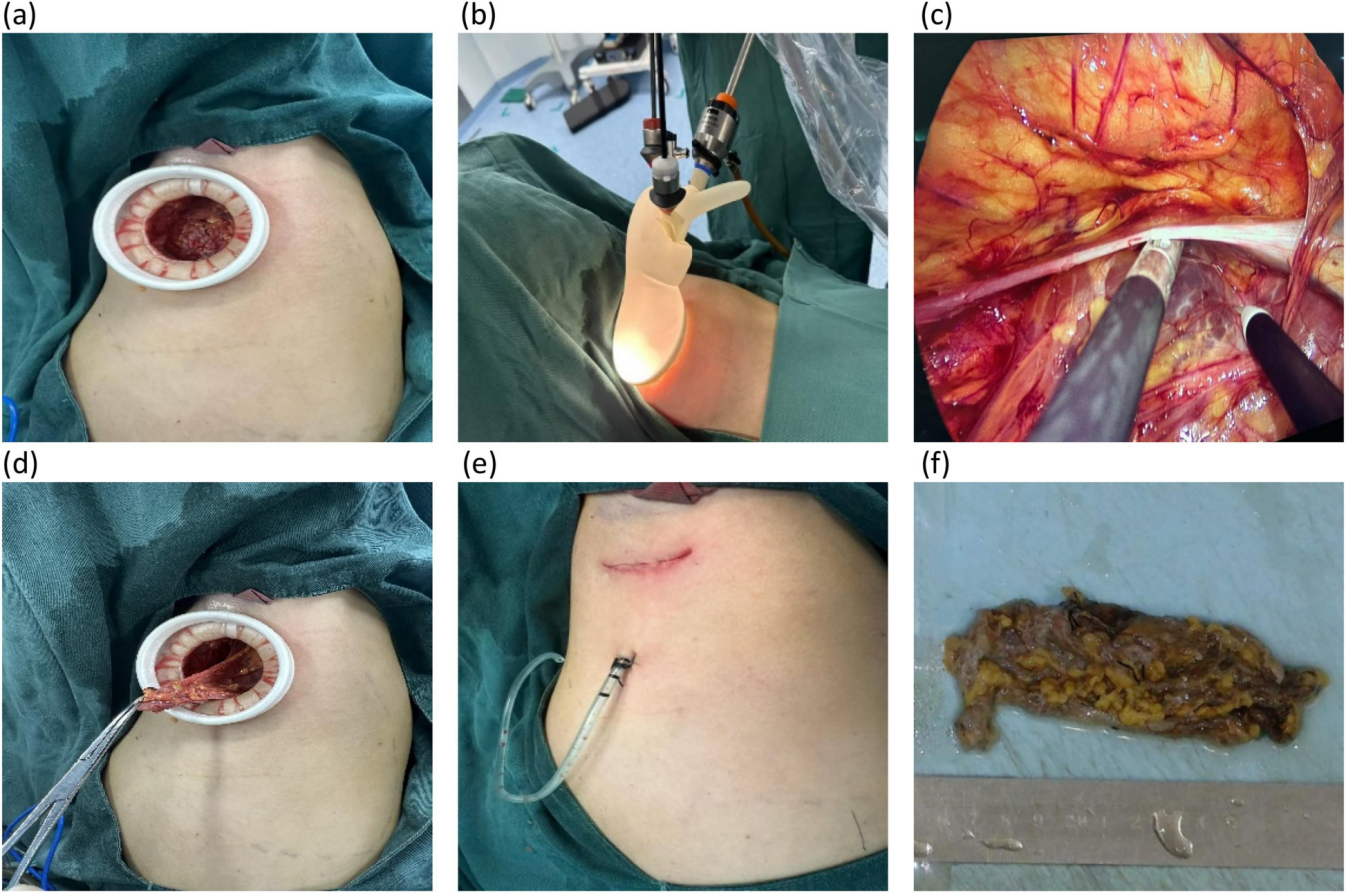
Intraoperative images. **(a)** Following the initial space dissection via a small incision, a wound protector is inserted. **(b)** An endoscopic working port is established through the wound protector. **(c)** A meticulous endoscopic dissection of the mass is performed. **(d)** The procedure is then converted to open exposure for the careful dissection of the axillary artery, axillary vein, and brachial plexus, followed by *en bloc* resection of the mass. **(e)** The incision is closed with a continuous intradermal suture, and a closed suction drain is placed. **(f)** The completely resected specimen is presented.

**Figure 3:**
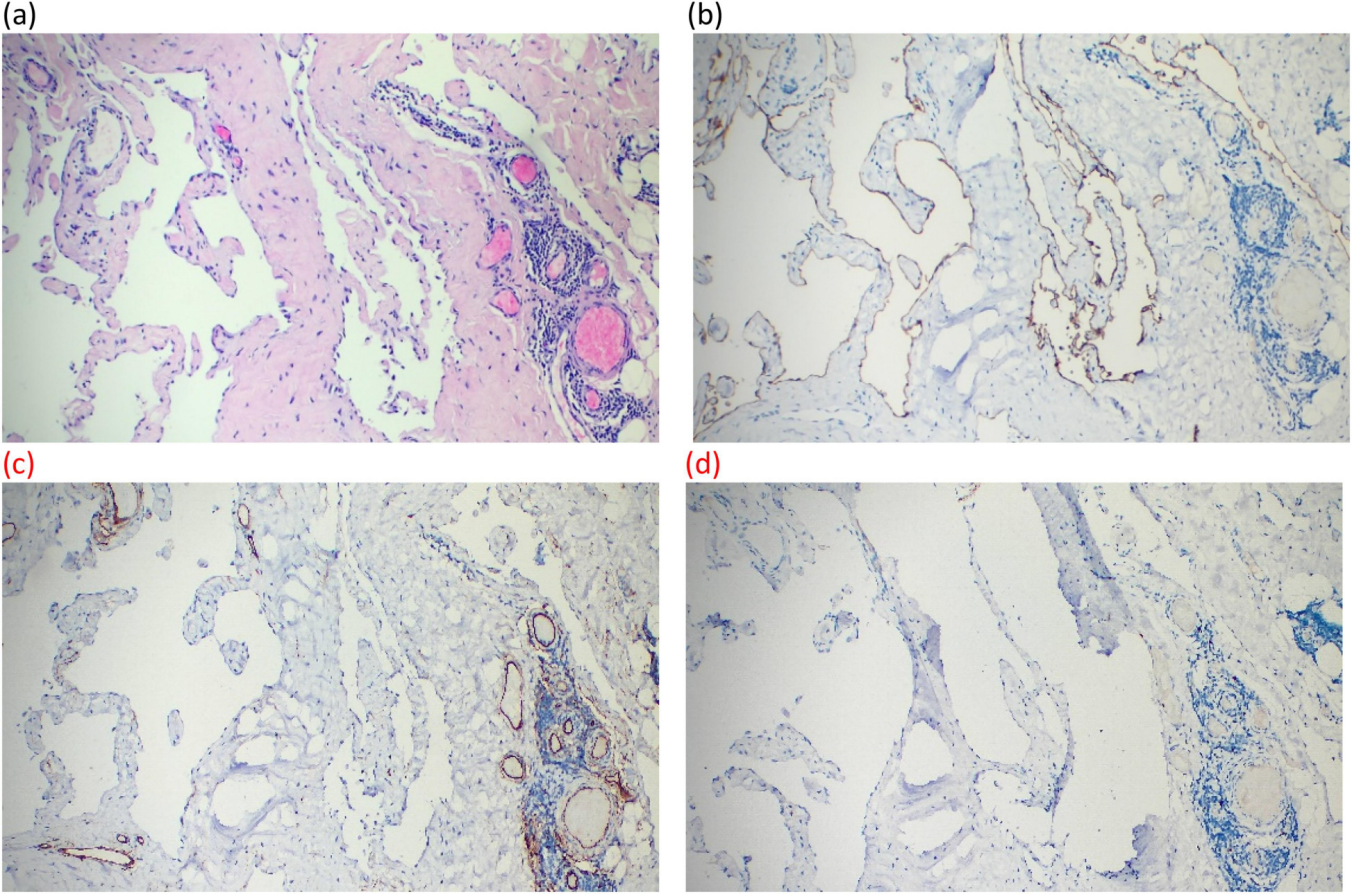
Postoperative pathology images. **(a)** Large cystic spaces are shown to be lined by flattened endothelium in HE staining (×10). **(b–d)** Lymphatic endothelial cells lining the cystic structures are shown to exhibit strong cytoplasmic positivity for D2-40 and negativity for CD34 and WT1 in sequence in immunohistochemical staining (×10).

On day 3 in the hospital, the patient underwent endoscopy-assisted excision of the giant, left axillary cystic lymphangioma (Figure 2). The procedure duration was 80 min with an estimated blood loss of 10 mL. The detailed surgical procedure is as follows. Under general anesthesia, the patient was placed in the right lateral decubitus position, with her left arm elevated and slightly abducted. A 4-cm transverse incision was made below the axillary crease. Subcutaneous dissection exposed the cystic mass, which appeared relatively distinct from the adjacent tissues. Blunt and sharp dissection freed the mass circumferentially, to approximately 3 cm from the incision. Approximately 300 mL of clear, pale yellow fluid was aspirated. A 50/60-mm wound protector was inserted through the incision and connected externally to a modified surgical glove. Three trocars (one 10-mm and two 5-mm) were placed through openings in the glove. Carbon dioxide insufflation was initiated, maintaining a pressure of 12 mmHg. A 30-degree endoscope, atraumatic grasper, and electrocautery hook were introduced. Endoscopic dissection continued circumferentially around the mass. The following key anatomical boundaries were identified and preserved: superiorly, the axillary artery and vein and brachial plexus; superomedially, the subclavian vessels and lateral thoracic vein; inferiorly, the level of the seventh rib (posterior axillary line); and laterally, the lateral border of the left scapula. Following insufflation release and instrument removal, the cyst was meticulously dissected free from the axillary vessels and adjacent nerves/lymphatics under direct vision and completely excised. The operative field was irrigated. A closed suction drain was placed. The incision was closed in layers and dressed, with the axilla lightly compressed. The histopathological examination confirmed that the mass was a cystic lymphangioma. Immunohistochemistry (IHC) showed the following: CD34(−), D2-40(+), WT1(−), and Ki-67 (1%) (Figure 3).

Oral intake resumed 4 h postoperatively. The drain was removed at 72 h. The patient was discharged on postoperative day 5. The 6-month follow-up chest CT showed no abnormalities, and the patient reported no symptoms.

## Discussion

Lymphangiomas are rare, with an estimated incidence of 1.2–2.8 per 100,000 (11). They can occur at any age but are more common in children (12–14). Histologically, they are classified into three types, namely, simple (capillary), cavernous, and cystic (hygroma) (15). Grossly, they appear as unilocular or multilocular cystic masses containing clear or chylous fluid. The present case exhibited a unilocular cyst with clear, pale yellow fluid. Routine histology can be challenging and immunohistochemistry is valuable, with D2-40 positivity strongly supporting a lymphatic endothelial origin (16). The patient's immunoprofile was characteristic (D2-40-positive and CD34- and WT1-negative). The majority of lymphangiomas are asymptomatic and discovered incidentally (17). Symptoms such as pain may arise from complications such as infection, hemorrhage, or a mass effect (17). The patient presented with a 1-month history of a painless axillary mass. The preoperative diagnosis relies on ultrasound, CT, and MRI (18, 19). Ultrasound typically shows an anechoic, cystic mass without internal vascularity, though flow may be detected in the septa/walls. High-resolution color Doppler ultrasound is highly sensitive for determining the size, location, and relationship of the mass. CT reveals a well-defined, round/oval, cystic mass with near-water density (CT value 0–20 HU), sometimes exhibiting a “crawling” growth pattern. Enhancement is absent in the cyst fluid and thin rim enhancement may occur. MRI clearly delineates the lesion as a well-circumscribed cyst with homogeneous long T1 and long T2 signal intensity and thin walls.

Surgical excision is the definitive treatment (6, 7, 20). Traditional open surgery allows for direct visualization and tactile feedback, which is advantageous for complex lesions intimately associated with neurovascular structures. However, it requires larger incisions, resulting in greater postoperative pain, longer scars, and potentially extended recovery times. Recurrence rates following open excision have been reported to range from 5% to 15% and can reach as high as 40% in cases with residual disease (21–23). Endoscopic approaches have been established for intra-abdominal, intrathoracic (24–26), and mediastinal lymphangiomas but have not been reported for axillary lesions. Purely endoscopic procedures are not suitable for all axillary lymphangiomas, particularly those with severe adhesions or large cysts. The endoscopy-assisted technique described in this case is a minimally invasive alternative, with the major advantages including (1) the accurate creation of the working space, utilizing a small initial open incision to establish the operative space and assess the tissue planes before transitioning to endoscopic dissection; (2) enhanced visualization, as the magnified endoscopic view during the pneumodissection provides vision of the anatomical layers with exceptional clarity, which is critical when dissecting around vital structures such as the brachial plexus and subclavian vessels; (3) improved cosmesis, as the use of small incisions represents a significant advantage over large open wounds, especially in younger patients; and (4) a potential reduction in complications, as minimal tissue trauma may translate into less postoperative pain and quicker recovery. A key drawback is the risk of cyst rupture during endoscopic manipulation within the confined, non-anatomical space of the axilla, which could potentially lead to recurrence. This hybrid technique, which combines the cosmetic and visual benefits of endoscopy with the safety and practicality of open exposure for the most critical steps of the dissection, facilitated complete resection and an uncomplicated outcome in this case. Lymphangiomas generally have a favorable prognosis, though recurrence is possible (27). Ultrasound or CT is suitable for follow-up. The patient remained recurrence-free at 6 months.

## Conclusion

This report presents the first successful case of endoscopy-assisted surgical resection of a giant, left axillary cystic lymphangioma. This technique uses a small incision for the initial access and workspace creation and combines this with endoscopic magnification for precise visualization of the critical anatomy, enabling safe and complete lesion removal. It offers the advantages of minimal invasiveness, rapid recovery, and favorable cosmesis. The absence of recurrence at the 6-month follow-up supports its efficacy and provides a valuable reference for managing similar axillary lesions.

## Data Availability

The original contributions presented in the study are included in the article/Supplementary Material, further inquiries can be directed to the corresponding author.
